# Dysfunctional BMPR2 signaling drives an abnormal endothelial requirement for glutamine in pulmonary arterial hypertension

**DOI:** 10.1086/690236

**Published:** 2017-02-01

**Authors:** Robert A. Egnatchik, Evan L. Brittain, Amy T. Shah, Wassim H. Fares, H. James Ford, Ken Monahan, Christie J. Kang, Emily G. Kocurek, Shijun Zhu, Thong Luong, Thuy T. Nguyen, Erik Hysinger, Eric D. Austin, Melissa C. Skala, Jamey D. Young, L. Jackson Roberts, Anna R. Hemnes, James West, Joshua P. Fessel

**Affiliations:** 1 Children’s Medical Center Research Institute, University of Texas Southwestern, Dallas, TX, USA; 2 Department of Chemical and Biomolecular Engineering, Vanderbilt University, Nashville, TN, USA; 3 Division of Cardiovascular Medicine and the Vanderbilt Translational and Clinical Cardiovascular Center, Department of Medicine, Vanderbilt University Medical Center, Nashville, TN, USA; 4 Department of Biomedical Engineering, Vanderbilt University, Nashville, TN, USA; 5 Section of Pulmonary, Critical Care & Sleep Medicine, Department of Medicine, Yale University, New Haven, CT, USA; 6 Division of Pulmonary Diseases and Critical Care Medicine, Department of Medicine, University of North Carolina Chapel Hill, Chapel Hill, NC, USA; 7 Division of Allergy, Pulmonary and Critical Care Medicine, Department of Medicine, Vanderbilt University Medical Center, Nashville, TN, USA; 8 Department of Pharmacology, Vanderbilt University, Nashville, TN, USA; 9 Division of Pulmonary Medicine, Children’s Hospital of Philadelphia, Philadelphia, PA, USA; 10 Division of Pulmonary Medicine, Department of Pediatrics, Vanderbilt University Medical Center, Nashville, TN, USA; 11 Department of Cancer Biology, Vanderbilt University, Nashville, TN, USA; 12 Department of Molecular Physiology and Biophysics, Vanderbilt University, Nashville, TN, USA

**Keywords:** glutaminolysis, metabolic reprogramming, mitochondria, bone morphogenic protein receptor type 2 (BMPR2), tricarboxylic acid (TCA) cycle

## Abstract

Pulmonary arterial hypertension (PAH) is increasingly recognized as a systemic disease driven by alteration in the normal functioning of multiple metabolic pathways affecting all of the major carbon substrates, including amino acids. We found that human pulmonary hypertension patients (WHO Group I, PAH) exhibit systemic and pulmonary-specific alterations in glutamine metabolism, with the diseased pulmonary vasculature taking up significantly more glutamine than that of controls. Using cell culture models and transgenic mice expressing PAH-causing BMPR2 mutations, we found that the pulmonary endothelium in PAH shunts significantly more glutamine carbon into the tricarboxylic acid (TCA) cycle than wild-type endothelium. Increased glutamine metabolism through the TCA cycle is required by the endothelium in PAH to survive, to sustain normal energetics, and to manifest the hyperproliferative phenotype characteristic of disease. The strict requirement for glutamine is driven by loss of sirtuin-3 (SIRT3) activity through covalent modification by reactive products of lipid peroxidation. Using 2-hydroxybenzylamine, a scavenger of reactive lipid peroxidation products, we were able to preserve SIRT3 function, to normalize glutamine metabolism, and to prevent the development of PAH in BMPR2 mutant mice. In PAH, targeting glutamine metabolism and the mechanisms that underlie glutamine-driven metabolic reprogramming represent a viable novel avenue for the development of potentially disease-modifying therapeutics that could be rapidly translated to human studies.

Alterations in the normal metabolic strategies utilized by various cell types are increasingly recognized as part of a central pathogenic mechanism in pulmonary arterial hypertension (PAH).^1,2^ Any given cell type—endothelium, smooth muscle, myocardium, skeletal muscle, etc.—exhibits a “metabolic program” under healthy, homeostatic conditions that is the sum total of the use and fate of all of the available carbon sources (primarily carbohydrates, fats, and amino acids). The details of a cell’s metabolic program are often particular for that cell type. For example, under normal conditions, cardiac myocytes primarily oxidize fatty acids as an energy source, whereas endothelial cells preferentially use glucose through oxidative and non-oxidative pathways.^3,4^ Any perturbation that places demands upon a cell to increase energy production, to increase macromolecule synthesis, or to resist pro-death stimuli will place a strain on the cell’s carbon resources and necessarily change the cell’s metabolic program. Conversely, anything that restricts a cell’s ability to use one or more carbon substrates can induce a metabolic reprogramming that will often change one or more fundamental properties of the cell, such as differentiation state, proliferative rate, or sensitivity to apoptosis. Thus, a cell’s metabolic program is inextricably linked to the role that cell plays in health and disease.

In PAH, it is well recognized that multiple cell types involved in disease pathogenesis exhibit a metabolic reprogramming characterized by increased shunting of glucose-derived carbon into non-oxidative, lactate-producing pathways in spite of the presence of ample oxygen supply to permit oxidative glucose metabolism.^1,2,5–7^ This is colloquially referred to as the “Warburg effect,” first described by Otto Warburg as a feature of cancer cells. However, the network of metabolic pathways within a cell is highly interconnected and it is rare for one pathway to be altered in isolation. Indeed, it is increasingly recognized that fatty acid metabolism is also markedly altered in PAH and that the reciprocal relationship between glucose and fatty acid oxidation (the so-called “Randle cycle”) is abnormal in PAH and likely contributes to pathogenesis in both the heart and in the pulmonary vasculature.^8–13^ The third major cellular carbon source—amino acids generally, and glutamine specifically—remains relatively understudied in PAH.^14^ Though amino acids represent the third major carbon source used by most cells, amino acid trafficking has mainly been studied in PAH in the context of nitric oxide synthesis. Recent discoveries in cancer biology have placed amino acids generally, and glutamine specifically, in central roles for biosynthesis, cellular energetics, and redox homeostasis.^15–18^ In the present study, we sought to examine glutamine metabolism in PAH in the specific context of dysfunctional signaling through bone morphogenic protein receptor type 2 (BMPR2). We hypothesized that the pulmonary endothelium in PAH would exhibit an abnormal increase in glutamine metabolism as a primary carbon source, in a manner similar to what has been observed in cancer.

## Materials and methods

### Reagents

^13^C_5_-L-glutamine was purchased from Sigma-Aldrich (St. Louis, MO, USA). Chetomin was purchased from Cayman Chemical (Ann Arbor, MI, USA). 2-hydroxybenzylamine (2HOBA) was synthesized at Vanderbilt as previously described.^19^ Antibodies were purchased as follows: HIF1α and glutamine synthetase, Novus Biologicals (Littleton, CO, USA); Sirt3 and α-tubulin, Cell Signaling Technology (Danvers, MA, USA); acetyl-lysine, EMD Millipore (Billerica, MA, USA); and Cox4 and histone H3, Abcam (Cambridge, MA, USA). Recombinant human SIRT3 was purchased from R&D Systems (Minneapolis, MN, USA). Sirt-Glo assay kit was purchased from Promega (Madison, WI, USA) and used according to the manufacturer’s instructions.

### Cell culture

We used previously characterized wild-type (WT) and BMPR2 mutant (BMPR2^R899X^) pulmonary microvascular endothelial cells (PMVECs) isolated from conditionally immortalized murine lines generated on the ImmortoMouse background,^20^ and WT and BMPR2 mutant PMVECs from a parent immortalized human line stably expressing either a WT or BMPR2 mutant construct transfected into the parent line and maintained under selection with G418S. For murine lines, cells were reverted to a primary endothelial phenotype by removal of murine interferon-gamma and transition to 37C (from 33C, which maintains conditional immortal phenotype through expression of SV40 large T), and doxycycline (300 ng/mL) was added to the media to induce expression of the transgene (the construct being Rosa26-rtTA x TetO_7_-Bmpr2^R899X^, the same as that used in Animal Studies), both for at least 72 h prior to experiments. Human lines stably express WT or mutant BMPR2, and selection with G418S was discontinued for 24 h prior to experiments.

### Metabolic fluxes and stable isotope labeling

Glucose and lactate levels were measured using the YSI 2300 Stat Glucose and Lactate Analyzer (Yellow Springs, OH, USA). High performance liquid chromatography (HPLC) was used to quantify amino acid concentrations with norvaline as an internal standard. Amino acid samples were then injected onto a Zorbax Eclipse Plus C18 column (Agilent) using a two-phase chromatography method as previously described.^21^ For isotope tracer studies, WT and BMPR2 mutant PMVECs were cultured in media containing 2 mM [U-^13^C_5_]-glutamine in place of unlabeled glutamine for 24 h. Analytes were extracted into ice-cold methanol and separated in 1:1 chloroform:H_2_O. The aqueous phase containing the amino and organic acids was then dried under air at room temperature. The samples were derivatized using MBTSTFA + 1% TBDMCS (Pierce). A total of 2 µL of each derivatized sample was then injected onto 30 m DB-35ms capillary column in an Agilent 6890N/5975B GC-MS. Flux rates were calculated using the ETA software package.^22^

### Cell proliferation assays

Cells were seeded and grown under specified media conditions as outlined in the “Results” section. Cells were counted for total and live cells using Trypan blue exclusion, and automated counts were done using a Countess cell counter (Life Technologies, Grand Island, NY, USA).

### Two-photon autofluorescence measurement of optical redox ratio

Two-photon images were acquired and analyzed as described previously.^23^ Briefly, cells were plated at a consistent density on glass-bottomed dishes and imaged 48 h later. A two-photon microscope (Bruker) and 40× oil-immersion objective (1.3 NA) were used to acquire NADH and FAD autofluorescence images for the same fields of view. Images were imported into MATLAB (Mathworks) and the NADH intensity was divided by the FAD intensity for each pixel to calculate a redox ratio image. The redox ratio was averaged across each image.

### Mitochondrial functional studies

To assess mitochondrial respiration in cultured PMVECs, cells were seeded in growth media in a 96-well plate from Seahorse Biosciences (Bilerica, MA, USA) at a seeding density of 50,000 live cells per well. The following day, cells were washed and placed in Seahorse Assay Media supplemented with specific substrates (1 gm/L glucose, 2 mM L-glutamine). After equilibration, oxygen consumption rates were measured on a Seahorse XFe96 analyzer using the Mito Stress Test protocol.

For mitochondrial protein measurements, mitochondria were isolated from fresh murine liver tissue as previously described.^24^

### Animal studies

All animal studies were approved by the Vanderbilt IACUC. Male and female FVB/N mice were aged 10–16 weeks. BMPR2^R899X^ mice were generated and maintained as previously described.^24,25^ For 2HOBA studies, 1 gm/L was administered in the drinking water, with water bottles protected from light and water changes every 2–3 days.

Echocardiography was performed on anesthetized mice (isoflurane anesthesia) to determine cardiac output. Right ventricular systolic pressure (RVSP) was invasively measured as described, using bromoethanol as the anesthetic.^24,25^

### Human studies

All human studies were approved by the Institutional Review Boards of Vanderbilt University, the University of North Carolina at Chapel Hill, and Yale University. Informed consent was obtained from each patient. For measurements of circulating glutamine levels, blood samples were obtained by peripheral venipuncture, centrifuged to separate plasma, and stored at –80℃ until analysis. Amino acid profiles were quantified in Vanderbilt’s clinical chemistry laboratory using a standard CLIA-approved method. For transpulmonary measurements, samples were obtained at the time of clinically indicated diagnostic right heart catheterization as previously described.^26,27^ Patients were classified as having pulmonary hypertension (PH) based upon the results of invasive hemodynamic testing, with differences between WHO groups determined by clinical history.

### Statistical analyses

All statistics were performed using GraphPad Prism 6.0 or Microsoft Excel unless otherwise specified. For animal experiments, sample sizes were chosen based upon previous experiments with approximately 80% of transgenic animals showing RVSP > 30 mmHg. Animals were randomly assigned to treatment groups by cage. For all measurements, investigators were blind to treatments and genotype until data analysis. Data were analyzed using two-tailed t-test or two-way ANOVA with Tukey’s post-test, as indicated. Significance was set at α < 0.05 after correction for multiple comparisons.

## Results

### Glutamine metabolism is abnormal in human PAH patients

**Fig. 1:**
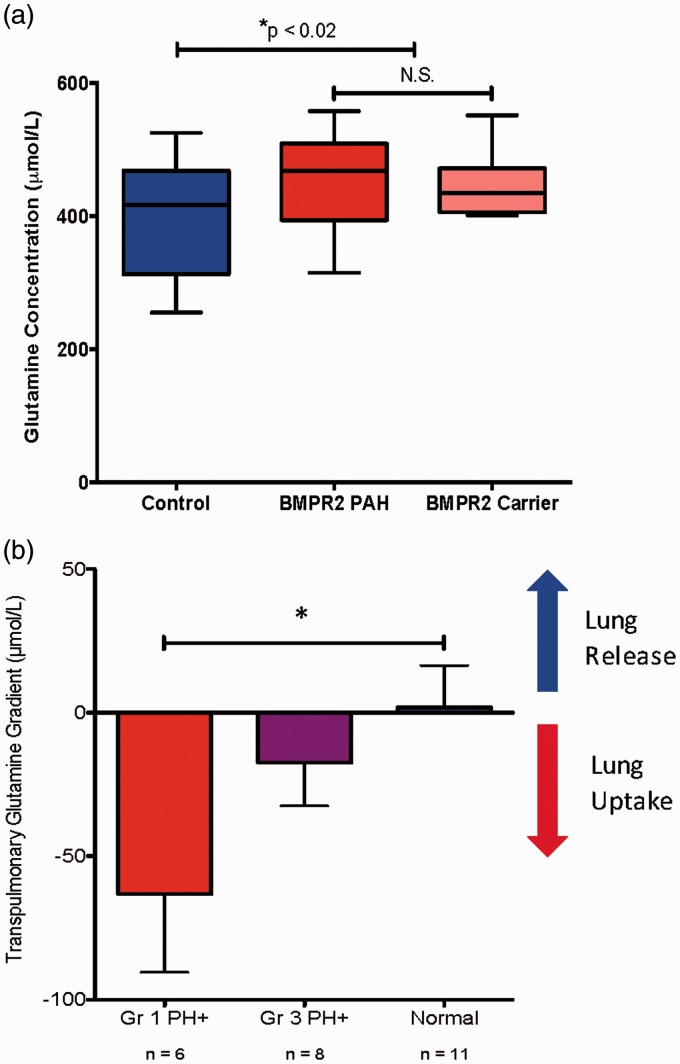
Humans with PAH and impaired BMPR2 signaling exhibit systemic and pulmonary vascular reprogramming of glutamine metabolism. (a) Humans with BMPR2 mutations, irrespective of the presence or absence of diagnosed PAH, have a statistically significant increase in circulating glutamine compared to family members with WT BMPR2. n = 11–24, *P < 0.02. (b) Transpulmonary glutamine uptake measured at right heart catheterization is significantly increased in patients with WHO Group I PAH compared to individuals with normal hemodynamics and to individuals with WHO Group III PAH. n = 6–11, *P < 0.05.

To test the hypothesis that PAH involves alteration of glutamine metabolism, we quantified fasting serum glutamine levels in heritable PAH patients with known BMPR2 mutations, in unaffected mutation carriers (individuals with known BMPR2 mutations but no evidence of PAH), and in married-in controls from the same households as patients and carriers. We found that circulating glutamine levels were significantly elevated in both heritable PAH patients (451 ± 68 umol/L) and in BMPR2 mutation carriers (450 ± 50 umol/L) compared to controls (399 ± 82  umol/L, *P* < 0.05, Fig. 1a). This was somewhat unexpected, as previous work has suggested increased cardiac glutamine uptake is a feature of the aberrant metabolic program in PAH.^14^ To better quantify glutamine metabolism within the pulmonary vasculature, we measured transpulmonary glutamine gradients in WHO Group I PAH patients, WHO Group III PH patients, and individuals with normal pulmonary hemodynamics. Samples were collected from the main pulmonary artery (PA) and from the pulmonary capillary wedge (PCW) position at the time of diagnostic right heart catheterization.^26^ Glutamine concentrations were quantified in each sample and the difference between the PCW and the PA samples for each individual was the gradient measurement (negative values indicate net uptake, positive values indicate net release). WHO Group I PAH patients showed substantial glutamine uptake by the pulmonary vasculature compared to WHO Group III patients and to controls (Fig. 1b). Taken together, these data indicated that PAH patients with abnormal BMPR2 function have marked changes in whole body and in pulmonary vascular glutamine metabolism.

### BMPR2 mutant endothelium exhibits abnormal avidity for glutamine carbon

**Fig. 2:**
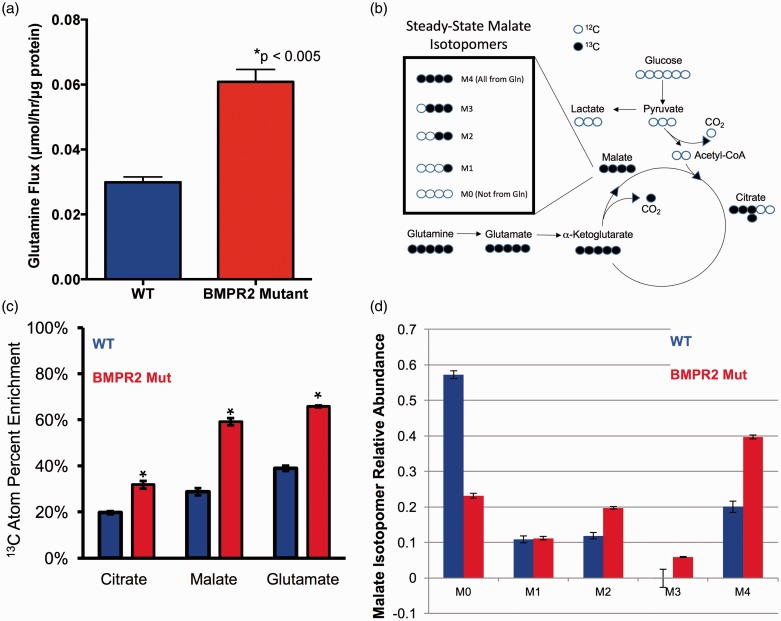
BMPR2 mutant PMVECs shuttle significantly more glutamine into the TCA cycle. (a) Specific extracellular glutamine uptake flux rates for BMPR2 mutant PMVECs are double those measured for WT PMVECs. n = 4 independent experiments, *P < 0.005 by two-tailed t-test. (b) Concept schematic for [U-^13^C_5_]-L-glutamine labeling to determine carbon flow into the TCA cycle. (c) Compared to WT, BMPR2 mutant PMVECs approximately double the amount of glutamine-derived carbon that is incorporated into multiple TCA cycle intermediates (citrate, malate, and glutamate, the immediate precursor for α-ketoglutarate) by ^13^C %atom enrichment quantified by mass spectrometry. n = 4, *P < 0.05 by two-way ANOVA with Tukey post-hoc test. (d) ^13^C_5_-L-glutamine labeling and quantification of TCA cycle intermediates by mass spectrometry shows a marked shift in the intracellular fate of glutamine-derived carbon in BMPR2 mutant PMVECs compared to WT, exemplified by the isotopomers of malate. In WT cells, the majority of TCA cycle carbon is unlabeled, represented by the M0 isotopomer, indicating use of an unlabeled carbon source (primarily glucose). By contrast, > 60% of the TCA carbon in the BMPR2 mutant PMVECs shows incorporation of at least two labeled carbons, with 40% of the total malate pool being fully labeled, represented by the M4 isotopomer. n = 3 each for WT and BMPR2 mutant.

We next sought to determine whether increased glutamine uptake by pulmonary endothelial cells with dysfunctional BMPR2 is an intrinsic property of those cells or merely due to the increased availability of glutamine. To assess this, we grew WT and BMPR2 mutant PMVECs in culture and provided glutamine in significant excess (2 mM) of physiologic levels in the culture media. Under conditions of glutamine excess, BMPR2 mutant PMVEC took up glutamine at twice the rate of WT cells (Fig. 2a), suggesting that increased glutamine uptake is intrinsic to PMVEC with dysfunctional BMPR2 signaling.

Glutamine can be used as a source of carbon input to the tricarboxylic acid (TCA) cycle, but it is also an important source of nitrogen in the cell, supplying nitrogen-requiring processes such as nucleotide synthesis. We hypothesized that glutamine was being used as a carbon source and that it was being preferentially shunted to the TCA cycle in BMPR2 mutant PMVEC compared to WT. To test this hypothesis, WT and BMPR2 mutant PMVEC were cultured for 24 h in the presence of 2 mM [U-^13^C_5_]-L-glutamine, a stable isotope of glutamine in which all five carbon atoms are carbon-13, which is easily detectable by mass spectrometry (see schematic Fig. 2b). The other major cellular carbon sources (glucose, fatty acids) were left unlabeled and the serum used had been dialyzed to remove free amino acids. Compared to WT, BMPR2 mutant PMVECs showed excess incorporation of glutamine-derived carbon in multiple intermediates of the TCA cycle (Fig. 2c). Furthermore, when specific TCA intermediates (e.g. malate) were broken down by tandem mass spectrometry to determine how many glutamine-derived carbon atoms had been incorporated (0, 1, 2, 3, or 4 for malate), we found that the majority of malate present (40%) contained four atoms of ^13^C, indicating that the majority of the carbon in the TCA cycle was coming from glutamine, as this was the sole source of ^13^C available (Fig. 2d). By contrast, in WT PMVECs, the majority of the malate present (>55%) contained no atoms of ^13^C, indicating that in WT cells, the majority of the carbon in the TCA cycle was not coming from glutamine but rather from the unlabeled glucose and fatty acids present.

### Glutamine is a required carbon source for BMPR2 mutant PMVECs

**Fig. 3:**
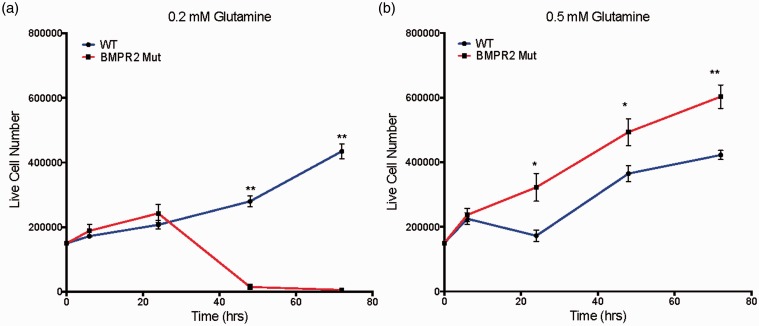
BMPR2 mutant PMVECs require increased glutamine availability to manifest hyperproliferative behavior typical of PAH. (a) In glutamine-limited conditions (0.2 mM), WT PMVECs are able to maintain steady proliferation, but BMPR2^R899X^ PMVEC begin to die rapidly after 24 h in culture. (b) In more glutamine-replete conditions (0.5 mM), WT PMVECs are essentially unaffected, whereas BMPR2^R899X^ PMVECs exhibit the hyperproliferative phenotype characteristic of PAH. n = 6 for each timepoint, *P < 0.01, **P < 0.001 by two-sided t-test.

We next wanted to determine whether BMPR2 mutant PMVECs have an absolute requirement for glutamine or whether this represented an “arrangement of convenience” as a result of the presence of excess glutamine. We cultured WT and BMPR2 mutant PMVECs in two concentrations of glutamine, 500 μM (mimicking serum concentrations in BMPR2 mutant patients) and 200 μM (representing the lowest levels that would be considered normal in humans). At 500 μM glutamine, BMPR2 mutant PMVECs show a net proliferation that exceeds the rate of WT PMVECs at all timepoints out to 72 h (Fig. 3a). At 200 μM glutamine, the net proliferative rate of WT PMVECs is essentially unchanged, but the BMPR2 mutant PMVECs are completely intolerant of glutamine-limited conditions and have all died by 72 h (Fig. 3b).

**Fig. 4:**
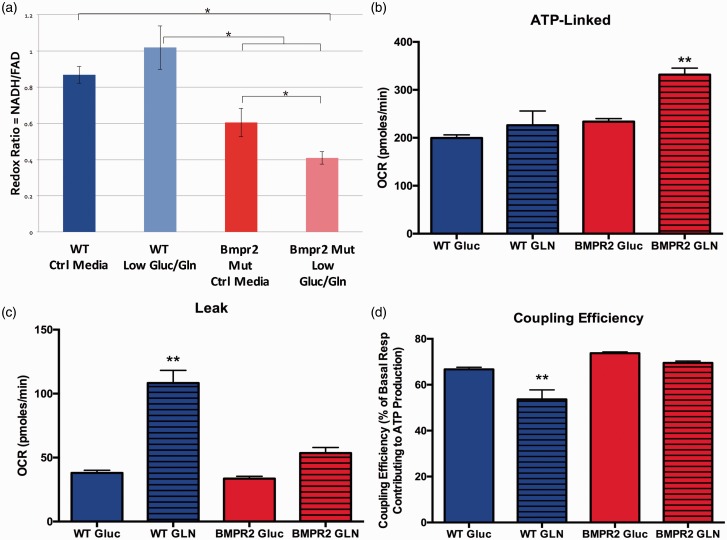
PAH-causing BMPR2 mutations drive mitochondrial dysfunction and metabolic reprogramming toward glutamine preference. (a) Quantifying intracellular NADH and FAD + pools in live cells using two-photon autofluorescence to calculate the optical redox ratio showed WT cultured human PMVECs retain metabolic flexibility by maintaining intracellular redox status despite limitation of the availability of glucose and glutamine as carbon sources. By contrast, PMVECs expressing mutant BMPR2 exhibit substantial reduction of redox ratio when glucose and glutamine are limited. n = 3 in standard media, n = 6 in glucose-free/glutamine-free media, *P < 0.05 by two-way ANOVA with Tukey post-hoc testing. (b–d) Compared to WT, cultured murine PMVECs expressing mutant Bmpr2 exhibit enhanced glutamine-supported ATP-linked mitochondrial respiration ((b) **P < 0.0001 by ANOVA vs. all other groups), decreased leak respiration ((c) **P < 0.0001 by ANOVA vs. all other groups), and increased coupling efficiency ((d), **P < 0.0001 by ANOVA vs. all other groups). n = 8–10 measurements for each condition per experiment, experiments performed in duplicate.

To better understand the details of glutamine utilization and the effects of glutamine limitation in BMPR2 mutant PMVECs, we quantified total intracellular redox status of WT and BMPR2 mutant PMVECs using two-photon autofluorescence of endogenous NADH and FAD, the major electron carriers controlled by TCA cycle activity. Fluorescence from both species allows calculation of the optical redox ratio, with a higher ratio indicating more TCA cycle activity, a more reduced intracellular redox environment, and increased overall metabolic activity. Conversely, a reduction in the optical redox ratio indicates an overall decrease in metabolic activity, particularly via the TCA cycle.^28–30^ Two-photon autofluorescence has very high time resolution, allowing for rapid changes in the metabolic and redox status of the cell to be detected and quantified. We quantified the optical redox ratio in WT and BMPR2 mutant PMVECs under basal conditions and with acute withdrawal of glutamine and glucose. At baseline, BMPR2 mutant PMVECs have a lower optical redox ratio than WT cells (Fig. 4a), indicating a relatively impaired ability to maintain the intracellular redox environment and relatively impaired overall metabolic activity. With acute limitation of TCA cycle substrates, WT PMVECs are able to rapidly adapt their metabolic behavior to maintain the optical redox ratio (Fig. 4a), whereas BMPR2 mutant PMVECs show a significant further reduction in the optical redox ratio (Fig. 4a), suggesting a significant loss of acute metabolic flexibility.

We next examined the consequences of TCA cycle glutamine dependence on mitochondrial function in BMPR2 mutant PMVECs. Cultured WT and BMPR2 mutant PMVECs were given either glucose or glutamine as the sole energy substrate available. Mitochondrial oxygen consumption was quantified using the Seahorse XFe96 analyzer and specific components of cellular respiration (ATP-linked respiration, proton leak, and coupling efficiency) were calculated from the oxygen consumption profiles (see Supplemental Figure 1 for schematic). WT PMVECs show equivalent ATP-linked respiration with glucose or glutamine as the sole carbon source, but BMPR2 mutant PMVECs show enhanced ATP-linked respiration with glutamine as their carbon source compared to glucose (Fig. 4b). When using glutamine, WT PMVECs exhibit increased proton leak in the mitochondria compared to when using glucose, but BMPR2 mutant cells maintain low levels of proton leak with either glutamine or glucose (Fig. 4c), suggesting that the overall efficiency for use of glutamine as a fuel to support ATP synthesis is much greater in BMPR2 mutant PMVECs compared to WT. Consistent with this, when comparing ATP-linked respiration to basal respiration, WT endothelial cells exhibited a lower coupling efficiency when using glutamine as fuel, whereas BMPR2 mutant PMVECs maintain a high coupling efficiency (Fig. 4d). Taken together, these data indicated that glutamine is the preferred substrate for energy production in BMPR2 mutant PMVECs.

### Oxidative stress drives HIF activation and SIRT3 impairment in BMPR2 mutants

**Fig. 5:**
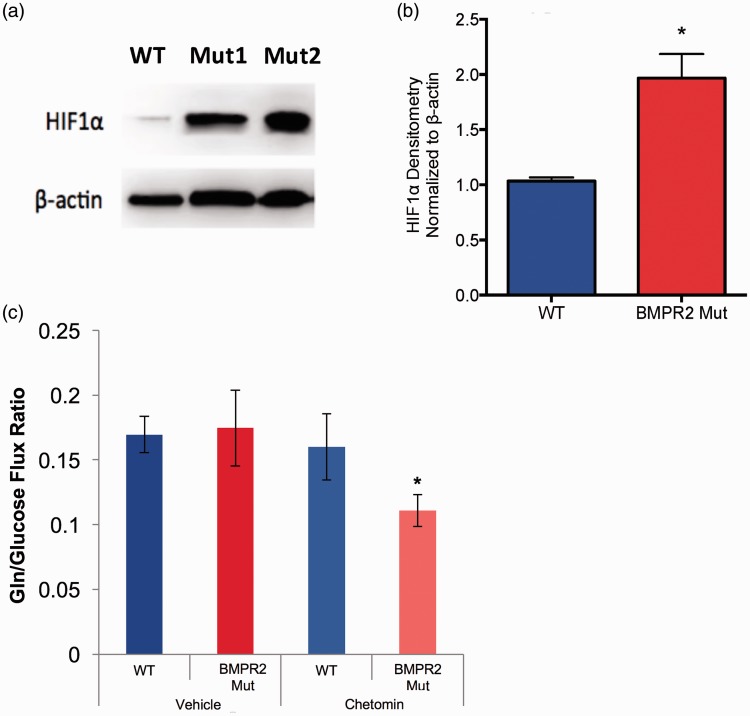
Normoxic HIF1α activation in BMPR2 mutant PMVEC contributes to metabolic reprogramming. (a, b) HIF1α is increased under normoxic conditions in two different types of BMPR2 mutations expressed in PMVEC (a) and is quantified by densitometry (b), n = 3 independent experiments each for human and murine PMVECs, data analyzed in aggregate with *P < 0.05 by two-tailed t-test. (c) Treatment of BMPR2 mutant PMVECs with low-dose chetomin, a pharmacologic inhibitor of HIF1α, significantly reduced the glutamine to glucose flux ratio while leaving WT PMVEC essentially unaffected. n = 3 for each condition, *P < 0.05.

We next wished to determine the molecular events downstream from BMPR2 mutation that contribute to glutamine reliance. Multiple signaling pathways have been shown to regulate increased glutamine utilization in a variety of disease contexts.^15,31–35^ Of the possible candidate pathways, the pathway with the greatest relevance to PAH that also potently regulates glutamine metabolism is hypoxia-inducible factor 1-alpha (HIF1α). HIF1α has been shown to be stabilized under normoxic conditions in experimental and human PAH, and activation of HIF1α can induce glutamine reliance.^36–39^ We found that BMPR2 mutant PMVECs grown in culture exhibited significant normoxic stabilization of HIF1α at the protein level compared to WT (Fig. 5a and 5b), and that this was demonstrable for two different PAH-associated BMPR2 mutation types. We then treated WT and BMPR2 mutant PMVECs with chetomin, a pharmacologic inhibitor of HIF, and assessed glucose and glutamine uptake by quantifying the extracellular flux ratio of glutamine to glucose. Treatment of WT cells had no effect on the glutamine to glucose flux ratio (Fig. 5c). However, treating BMPR2 mutant PMVECs with the HIF inhibitor significantly reduced the glutamine to glucose flux ratio (Fig. 5c), suggesting that HIF1α activity helps to drive the glutamine requirement in BMPR2 mutant endothelium.

Though HIF1α activation contributes to glutamine uptake and utilization in BMPR2 mutant cells, we suspected that there were additional alterations in signaling pathways known to control metabolism. Activation of HIF1α by itself has been shown to drive glutamine metabolism, but the glutamine is disproportionately used for biosynthesis.^39–41^ Given the finding that glutamine in BMPR2 mutant PMVECs is being used for energy production and not obviously for disproportionate biosynthesis, we hypothesized that a metabolic control pathway directly involved in both glutamine regulation and energy production was likely altered. Sirtuin-3 (SIRT3), a lysine deacetylase involved in mitochondrial energy production and redox homeostasis, emerged as a strong candidate. Loss of SIRT3 has been shown to lie upstream of HIF1 activation and has been associated with PH.^15,42–44^

**Fig. 6:**
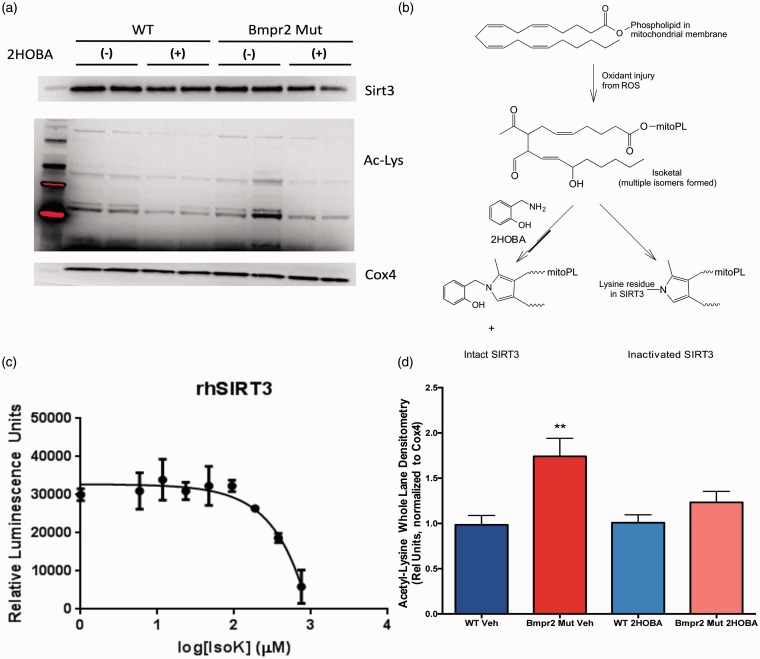
SIRT3 is inactivated in BMPR2^R899X^ mice. (a) Mitochondria isolated from BMPR2^R899X^ mice have equivalent SIRT3 content compared to WT, but exhibit hyperacetylation of multiple mitochondrial proteins (lanes 1–2 and 5–6), indicating loss of SIRT3 function. Treatment of BMPR2^R899X^ mice with 2HOBA, a scavenger of isoketals, normalized mitochondrial protein acetylation state (lanes 3–4 and 7–8). Western images are separate serial exposures of the same blot following stripping and reprobing with antibodies to the indicated proteins. (b) Schematic showing how oxidative injury to the mitochondrial membrane is thought to inactivate SIRT3 via adduction by isoketals. (c) Purified recombinant human SIRT3 activity measured by luminescence assay (Sirt-Glo, Promega) is dose-dependently inhibited by isoketals. (d) Densitometry quantification of 4–9 animals from experiments performed in triplicate. **P < 0.01 vs. all other groups by ANOVA with Tukey post-hoc testing.

Because sirtuin expression and activity is tightly regulated by substrate availability, nutrient intake, physical activity, and interacting signaling pathways operating between organ systems, the role of SIRT3 in BMPR2-mediated PAH is best studied in a murine model system. To investigate the role of SIRT3 inactivation in BMPR2-mediated PAH, we isolated mitochondria from WT and BMPR2 mutant (BMPR2^R899X^) mice fed a Western diet (60% calories from fat) for 8 weeks. Quality of the mitochondria-enriched fraction was confirmed (Supplemental Figure 2) by demonstrating the presence of COX4 and the absence of histone H3 (nuclear protein) and α-tubulin (cytosolic protein), and the mitochondrial proteome was assessed for acetylation of lysine residues, with SIRT3 inactivation leading to lysine hyperacetylation. Compared to WT, BMPR2^R899X^ mitochondria had equivalent protein levels of SIRT3 but exhibited significant lysine hyperacetylation of multiple mitochondrial proteins (Fig. 6a, lanes 1–2 for WT and 5–6 for mutants, quantified in Fig. 6d), consistent with loss of SIRT3 activity in BMPR2^R899X^ mice.

Hyperacetylation of the mitochondrial proteome with no change in SIRT3 protein content is consistent with loss of SIRT3 enzymatic activity. We have previously shown that the mitochondrial membranes specifically in BMPR2^R899X^ mice exhibit extensive lipid peroxidation as a function of expression of the mutant BMPR2 isoform, and that the lipid peroxidation is characterized by excess production of covalent adducts between proteins and isoketals, a subset of highly reactive lipid peroxidation products, specifically in the lung around the microvessels.^24,45^ We and others have also shown that lipid peroxidation products are increased in humans with PAH.^46–49^ We thus hypothesized that SIRT3 was undergoing oxidative inactivation in the mitochondria of BMPR2^R899X^ mice, and that treatment with the isoketal scavenger 2HOBA would restore normal SIRT3 activity (schematic in Fig. 6b). We demonstrated the biochemical plausibility of this hypothesis by incubating synthetically pure isoketal with recombinant human SIRT3 in vitro. Isoketal treatment inactivated SIRT3 in a concentration-dependent manner as measured by luminescence via the Sirt-Glo assay (Fig. 6c). We then directly tested the hypothesis that scavenging isoketals would preserve SIRT3 function by treating WT and BMPR2^R899X^ mice with 1 g/L 2HOBA in their drinking water. Treatment with 2HOBA significantly reduced lysine acetylation in the mitochondrial proteome in BMPR2^R899X^ mice compared to WT without affecting total SIRT3 content in the mitochondria (Fig. 6a, lanes 3–4 for WT and 7–8 for mutants, quantified in Fig. 6d), consistent with preservation of SIRT3 catalytic activity.

### 2HOBA normalizes glutamine metabolism and prevents PAH in vivo

**Fig. 7:**
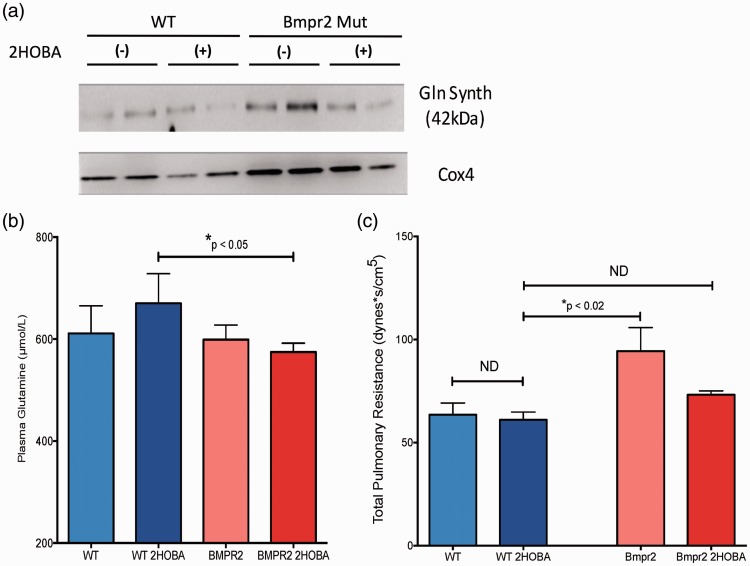
2HOBA lowers circulating glutamine and prevents the development of PAH in BMPR2^R899X^ mice. (a) Glutamine synthetase in the liver mitochondrial fraction is elevated in BMPR2^R899X^ mice (lanes 5–6) compared to WT (lanes 1–2). 2HOBA treatment reduced glutamine synthetase in the BMPR2^R899X^ mice (lanes 7–8) but not substantially in WT (lanes 3–4). Western images are separate serial exposures of the same blot following stripping and reprobing with antibodies to the indicated proteins. (b) Treatment with 2HOBA significantly reduces plasma glutamine availability in BMPR2^R899X^ mice compared to 2HOBA-treated WT mice. n = 6–29, *P < 0.05. (c) 2HOBA treatment reduced total pulmonary resistance in BMPR2^R899X^ mice to a level statistically indistinguishable from WT. n = 5–14, *P < 0.02 by ANOVA with Tukey post-hoc analysis.

Having shown that impaired BMPR2 function is associated with loss of SIRT3 function, and that this can be prevented with 2HOBA treatment to scavenge damaging lipid peroxidation products, we next wished to assess the in vivo metabolic dysfunction downstream from BMPR2 mutation, the relationship to PAH, and the effect of 2HOBA treatment. We hypothesized that 2HOBA would prevent the development of PAH in BMPR2^R899X^ mice and would have a beneficial modulatory effect on glutamine metabolism in vivo. Expression of the BMPR2^R899X^ allele was sufficient to drive upregulation of glutamine synthetase in skeletal muscle (Supplemental Figure 3) and in liver mitochondria (Fig. 7a) compared to WT. Despite upregulation of glutamine synthetase, circulating glutamine concentrations were equivalent in vehicle-treated WT and BMPR2^R899X^ mice (Fig. 7b), suggesting that increased glutamine synthesis was being matched by increased consumption in the BMPR2 mutants. Following 2HOBA treatment, BMPR2^R899X^ mice had reduced glutamine synthetase in liver mitochondria (Fig. 7a) and significantly lower circulating glutamine compared to treated WT mice (Fig. 7b), suggesting a favorable effect on glutamine balance in the mutants. Consistent with the effects on SIRT3 and glutamine metabolism, 2HOBA treatment prevented the development of PH as measured by total pulmonary resistance in the BMPR2^R899X^ mice compared to vehicle-treated mice (Fig. 7c). Treatment with 2HOBA in the BMPR2^R899X^ mice modestly increased cardiac output (Supplemental Figure 4a) and decreased RVSP (Supplemental Figure 4b), the combined effect of which was to significantly reduce total pulmonary resistance.

## Discussion

**Fig. 8:**
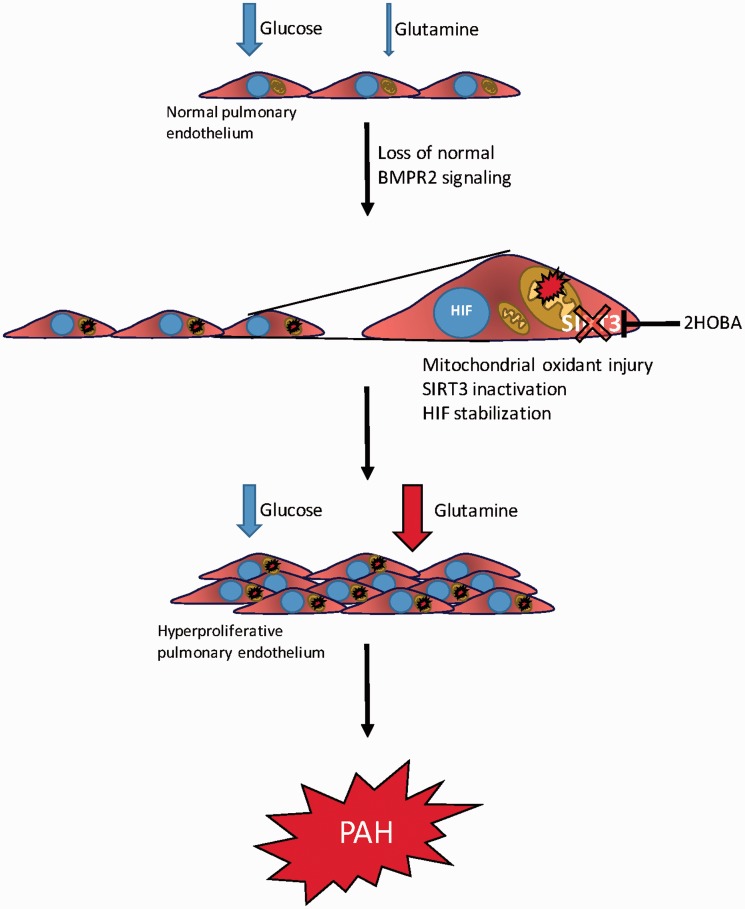
Schematic of BMPR2-mediated metabolic reprogramming. The normal pulmonary endothelium relies mainly on glucose as its bioenergetic fuel. When normal BMPR2 signaling is lost, oxidant injury in the mitochondria drives inactivation of SIRT3 via adduction by isoketals. This can be interrupted with 2HOBA. Unchecked, however, continued oxidant injury and SIRT3 inactivation (either in sequence or in parallel) lead to HIF stabilization, all of which drives a hyperproliferative, glutamine avid pulmonary endothelial phenotype that underlies the development of PAH.

In these studies, we have used human and murine cell culture models, transgenic mice, and samples from living PAH patients to demonstrate a markedly altered metabolic program for glutamine due to dysfunctional BMPR2 signaling (Fig. 8). Loss of normal BMPR2 function leads to oxidant injury in the mitochondria and formation of reactive products of lipid peroxidation termed isoketals.^45^ Isoketals inactivate SIRT3 which, together with increased oxidant stress, results in stabilization of HIF1α. Together, SIRT3 and HIF1α are two of the best established “master regulator” pathways for cellular metabolism generally and glutamine metabolism specifically. We show that this process can be interrupted in vivo by treating with an orally bioavailable scavenger of isoketals—2HOBA—and that interruption of the molecular cascade leading to glutamine addiction prevents the development of PAH.

Our findings add to the rapidly growing weight of evidence implicating altered cellular metabolism as a major contributor to the development and maintenance of PH. The first major metabolic perturbation described in PAH was a shift to lactate-producing glycolysis despite the presence of ample oxygen concentrations to permit complete glucose oxidation. This finding has now been replicated in virtually every setting of experimental and human PAH, and the molecular underpinnings continue to be clarified.^1,2,6,11,38,50–52^ However, as has also been true for cancer research, we and others are finding that the changes to the cellular metabolic landscape in PAH are not restricted to glycolysis, but rather appear to involve most of the major metabolic pathways. Given the interconnectedness of the metabolome in healthy cells, this is perhaps not surprising. Indeed, we have previously shown that expression of mutant isoforms of BMPR2 in PMVECs is sufficient to reprogram multiple metabolic pathways.^51^

Our findings help bring together several other recently published reports concerning the altered metabolic program in PH. Piao et al. showed increased right ventricular (RV) glutaminolysis in monocrotaline-treated rats, and that a signature of increased glutaminolysis was also present in the RV from human PAH patients.^14^ Paulin et al. recently published that loss of SIRT3 activity through reduction at the protein level predisposed mice to the development of PAH, though the mechanism of downregulation was not entirely clear.^44^ Extending these findings, Lai et al. have very recently shown an important role for SIRT3 in PH and the associated systemic metabolic derangements seen in left heart failure with preserved ejection fraction.^53^ Diebold et al. reported metabolic remodeling in pulmonary endothelial cells with reduced BMPR2 expression, though manifestation of the PAH phenotype required exposure to hypoxia, implicating HIF but not SIRT3.^50,54^ Our findings fill in important gaps within and between these studies by providing a possible mechanism by which all of these published findings could be consistent with one another. In addition, we provide evidence in living PAH patients that dynamic metabolism is in fact reprogrammed in a manner similar to what has been observed in model systems and postmortem tissue. We have also shown that targeting at least one of the mechanisms underlying metabolic reprogramming in PAH has beneficial effects in vivo, and we have done so with a compound that can be used in humans. 2HOBA has a very favorable long-term safety profile, with over 12 months of continuous dosing in mice producing no significant toxicity. 2HOBA is a naturally occurring product, allowing expedited proof-of-principle studies in humans, with subsequent studies focusing on related compounds with improved pharmacokinetics. Importantly, 2HOBA’s mechanism of action has been demonstrated in other disease models and across species.^19,55^

Our study does have some important limitations. We have hypothesized a mechanism in which extensive mitochondrial lipid peroxidation results in SIRT3 inactivation followed by HIF1 stabilization. While there are published data placing SIRT3 upstream of HIF1, there is also an extensive literature on oxidative stress by itself being sufficient to activate HIF. Our data could be consistent with concurrent inactivation of SIRT3 and stabilization of HIF, both being driven by oxidant injury. We were only able to quantify transpulmonary metabolite gradients in a small number of patients and these findings need expansion and independent replication. Our studies have focused on glutamine as a carbon source, which was necessary for acquiring a deeper understanding of how glutamine trafficking in the pulmonary endothelium becomes dysregulated in PAH. Indeed, even the findings regarding glutamine as a carbon source as we have presented them are likely only part of the story. Our own ^13^C labeling data indicate that complex carbon cycling through the pyruvate pool is occurring, as shown by the presence of the M3 isotopomer in the BMPR2 mutant PMVECs (Fig. 2d) and the presence of excess label incorporation in the alanine pool but not in the lactate pool (Supplemental Figure 5). This may be related to a degree of carbon flux through the pyruvate carboxylate pathway, as has been found in another recent study examining upregulation of vascular glutaminolysis in PAH.^56^ A deeper understanding of the utilization and trafficking of glutamine as a carbon source in PAH will be a goal of our ongoing studies, not only to address these questions, but also to address issues such as how changes in TCA intermediates such as succinate or 2-oxoglutarate (neither of which were quantified in the present study, but which very likely are impacted by changes in glutamine metabolism) may impact directly on HIF activation.^57,58^ Besides engaging in complex biochemistry as a carbon source, glutamine can also serve as an important nitrogen source, contributing to biosynthetic pathways for nucleic acids and amino acids as well as participation in the urea cycle and nitrogen cycling pathways. Our studies were not structured to capture this aspect of glutamine metabolism and this will likely be a fruitful avenue of inquiry in future studies. Our work deliberately did not address the use and fate of fat-derived carbon in BMPR2 mutant endothelium. Fatty acid carbon trafficking is much more important in normal endothelial biology than was previously realized.^59^ While we have previously addressed fatty acid metabolism in BMPR2 mutant tissues, and we have previously shown evidence for downregulation of key fat metabolism genes in BMPR2 mutant pulmonary endothelial cells, the detailed complexities of utilization of multiple carbon sources were beyond the scope of this report.^9,12,13,51^

In summary, we have shown that metabolic reprogramming in the pulmonary endothelium driving BMPR2-mediated PAH involves glutamine addiction as an important molecular feature. Glutamine becomes an indispensable, preferred carbon source in the BMPR2 mutant pulmonary endothelium, permitting hyperproliferation characteristic of PAH. Glutamine addiction appears to be driven downstream from BMPR2 by decreases in SIRT3 activity and increased HIF1α signaling. Interruption of this pathway in BMPR2 mutant mice with a molecularly targeted therapy that is directly translatable to humans in large part ameliorates disease. The finding that glutamine avidity is present in humans with WHO Group I PAH suggests that 2HOBA and other therapies targeting metabolic reprogramming have a high likelihood of benefit in PAH and delays to trials of these interventions in PAH patients should be minimized.

## References

[1] Sutendra G Michelakis ED The metabolic basis of pulmonary arterial hypertension. Cell Metab 2014; 19: 558–573. 24508506 10.1016/j.cmet.2014.01.004

[2] Ryan JJ Archer SL Emerging concepts in the molecular basis of pulmonary arterial hypertension: part I: metabolic plasticity and mitochondrial dynamics in the pulmonary circulation and right ventricle in pulmonary arterial hypertension. Circulation 2015; 131: 1691–1702. 25964279 10.1161/CIRCULATIONAHA.114.006979 PMC4429908

[3] Taegtmeyer H Young ME Lopaschuk GD Assessing cardiac metabolism: a scientific statement from the American Heart Association. Circ Res 2016; 118: 1659–1701. 27012580 10.1161/RES.0000000000000097 PMC5130157

[4] Eelen G de Zeeuw P Simons M Endothelial cell metabolism in normal and diseased vasculature. Circ Res 2015; 116: 1231–1244. 25814684 10.1161/CIRCRESAHA.116.302855 PMC4380230

[5] Gomez-Arroyo J Mizuno S Szczepanek K Metabolic gene remodeling and mitochondrial dysfunction in failing right ventricular hypertrophy secondary to pulmonary arterial hypertension. Circ Heart Fail 2013; 6: 136–144. 23152488 10.1161/CIRCHEARTFAILURE.111.966127 PMC3790960

[6] Ryan J Dasgupta A Huston J Mitochondrial dynamics in pulmonary arterial hypertension. J Mol Med 2015; 93: 229–242. 25672499 10.1007/s00109-015-1263-5 PMC4339102

[7] Obre E Rossignol R Emerging concepts in bioenergetics and cancer research: metabolic flexibility, coupling, symbiosis, switch, oxidative tumors, metabolic remodeling, signaling and bioenergetic therapy. Int J Biochem Cell Biol 2015; 59: 167–181. 25542180 10.1016/j.biocel.2014.12.008

[8] Chen X Talati M Fessel JP Estrogen metabolite 16alpha-hydroxyestrone exacerbates bone morphogenetic protein receptor type II-associated pulmonary arterial hypertension through microRNA-29-mediated modulation of cellular metabolism. Circulation 2016; 133: 82–97. 26487756 10.1161/CIRCULATIONAHA.115.016133 PMC4698046

[9] Hemnes AR Brittain EL Trammell AW Evidence for right ventricular lipotoxicity in heritable pulmonary arterial hypertension. Am J Respir Crit Care Med 2014; 189: 325–334. 24274756 10.1164/rccm.201306-1086OC PMC3977729

[10] West J Niswender KD Johnson JA A potential role for insulin resistance in experimental pulmonary hypertension. Eur Respir J 2013; 41: 861–871. 22936709 10.1183/09031936.00030312 PMC3746982

[11] Dyck JR Hopkins TA Bonnet S Absence of malonyl coenzyme A decarboxylase in mice increases cardiac glucose oxidation and protects the heart from ischemic injury. Circulation 2006; 114: 1721–1728. 17030679 10.1161/CIRCULATIONAHA.106.642009

[12] Talati MH Brittain EL Fessel JP Mechanisms of lipid accumulation in the bone morphogenic protein receptor 2 mutant right ventricle. Am J Respir Crit Care Med 2016; 194: 719–728. 27077479 10.1164/rccm.201507-1444OC PMC5027228

[13] Brittain EL Talati M Fessel JP Fatty acid metabolic defects and right ventricular lipotoxicity in human pulmonary arterial hypertension. Circulation 2016; 133: 1936–1944. 27006481 10.1161/CIRCULATIONAHA.115.019351 PMC4870107

[14] Piao L Fang YH Parikh K Cardiac glutaminolysis: a maladaptive cancer metabolism pathway in the right ventricle in pulmonary hypertension. J Mol Med 2013; 91: 1185–1197. 23794090 10.1007/s00109-013-1064-7 PMC3783571

[15] Li C Zhang G Zhao L Metabolic reprogramming in cancer cells: glycolysis, glutaminolysis, and Bcl-2 proteins as novel therapeutic targets for cancer. World J Surg Oncol 2016; 14: 15. 26791262 10.1186/s12957-016-0769-9 PMC4721116

[16] Yang C Ko B Hensley CT Glutamine oxidation maintains the TCA cycle and cell survival during impaired mitochondrial pyruvate transport. Mol Cell 2014; 56: 414–424. 25458842 10.1016/j.molcel.2014.09.025 PMC4268166

[17] Jiang L Shestov AA Swain P Reductive carboxylation supports redox homeostasis during anchorage-independent growth. Nature 2016; 532: 255–258. 27049945 10.1038/nature17393 PMC4860952

[18] DeBerardinis RJ Chandel NS Fundamentals of cancer metabolism. Sci Adv 2016; 2: e1600200. 27386546 10.1126/sciadv.1600200 PMC4928883

[19] Kirabo A Fontana V de Faria AP DC isoketal-modified proteins activate T cells and promote hypertension. J Clin Invest 2014; 124: 4642–4656. 25244096 10.1172/JCI74084 PMC4220659

[20] Majka S Hagen M Blackwell T Physiologic and molecular consequences of endothelial Bmpr2 mutation. Respir Res 2011; 12: 84. 21696628 10.1186/1465-9921-12-84 PMC3141420

[21] Greene J Henderson JW Wikswo JP Rapid and precise determination of cellular amino acid flux rates using HPLC with automated derivatization with absorbance detection, Wilmington, DE: Agilent Technologies, 2009.

[22] Murphy TA Young JD ETA: robust software for determination of cell specific rates from extracellular time courses. Biotechnol Bioeng 2013; 110: 1748–1758. 23296385 10.1002/bit.24836 PMC3863648

[23] Shah AT Demory Beckler M Walsh AJ Optical metabolic imaging of treatment response in human head and neck squamous cell carcinoma. PLoS One 2014; 9: e90746. 24595244 10.1371/journal.pone.0090746 PMC3942493

[24] Fessel JP Flynn CR Robinson LJ Hyperoxia synergizes with mutant bone morphogenic protein receptor 2 to cause metabolic stress, oxidant injury, and pulmonary hypertension. Am J Respir Cell Mol Biol 2013; 49: 778–787. 23742019 10.1165/rcmb.2012-0463OC PMC3931097

[25] Johnson JA Hemnes AR Perrien DS Cytoskeletal defects in Bmpr2-associated pulmonary arterial hypertension. Am J Physiol Lung Cell Mol Physiol 2012; 302: L474–484. 22180660 10.1152/ajplung.00202.2011 PMC3311512

[26] Fares WH Ford HJ Ghio AJ Safety and feasibility of obtaining wedged pulmonary artery samples and differential distribution of biomarkers in pulmonary hypertension. Pulm Circ 2012; 2: 477–482. 23372932 10.4103/2045-8932.105036 PMC3555418

[27] Monahan K Scott TA Su YR Reproducibility of intracardiac and transpulmonary biomarkers in the evaluation of pulmonary hypertension. Pulm Circ 2013; 3: 345–349. 24015334 10.4103/2045-8932.114762 PMC3757828

[28] Skala MC Riching KM Gendron-Fitzpatrick A In vivo multiphoton microscopy of NADH and FAD redox states, fluorescence lifetimes, and cellular morphology in precancerous epithelia. Proc Natl Acad Sci U S A 2007; 104: 19494–19499. 18042710 10.1073/pnas.0708425104 PMC2148317

[29] Chance B Schoener B Oshino R Oxidation-reduction ratio studies of mitochondria in freeze-trapped samples. NADH and flavoprotein fluorescence signals. J Biol Chem 1979; 254: 4764–4771. 220260

[30] Barlow CH Harden WR 3rd Harken AH Fluorescence mapping of mitochondrial redox changes in heart and brain. Crit Care Med 1979; 7: 402–406. 223813 10.1097/00003246-197909000-00011

[31] Sanchez EL Lagunoff M Viral activation of cellular metabolism. Virology 2015; 479–480: 609–618. 10.1016/j.virol.2015.02.038 PMC4424078 25812764

[32] Hough KP Chisolm DA Weinmann AS Transcriptional regulation of T cell metabolism. Mol Immunol 2015; 68: 520–526. 26298576 10.1016/j.molimm.2015.07.038 PMC4679508

[33] Courtnay R Ngo DC Malik N Cancer metabolism and the Warburg effect: the role of HIF-1 and PI3K. Mol Biol Rep 2015; 42: 841–851. 25689954 10.1007/s11033-015-3858-x

[34] Ryan JJ Archer SL The right ventricle in pulmonary arterial hypertension: disorders of metabolism, angiogenesis and adrenergic signaling in right ventricular failure. Circ Res 2014; 115: 176–188. 24951766 10.1161/CIRCRESAHA.113.301129 PMC4112290

[35] Chen JQ Russo J Dysregulation of glucose transport, glycolysis, TCA cycle and glutaminolysis by oncogenes and tumor suppressors in cancer cells. Biochim Biophys Acta 2012; 1826: 370–384. 22750268 10.1016/j.bbcan.2012.06.004

[36] Tuder RM Archer SL Dorfmuller P Relevant issues in the pathology and pathobiology of pulmonary hypertension. J Am Coll Cardiol 2013; 62: D4–12. 24355640 10.1016/j.jacc.2013.10.025 PMC3970402

[37] Pisarcik S Maylor J Lu W Activation of hypoxia-inducible factor-1 in pulmonary arterial smooth muscle cells by endothelin-1. Am J Physiol Lung Cell Mol Physiol 2013; 304: L549–561. 23418090 10.1152/ajplung.00081.2012 PMC3625988

[38] Fijalkowska I Xu W Comhair SA Hypoxia inducible-factor1alpha regulates the metabolic shift of pulmonary hypertensive endothelial cells. Am J Pathol 2010; 176: 1130–1138. 20110409 10.2353/ajpath.2010.090832 PMC2832136

[39] Wise DR Ward PS Shay JE Hypoxia promotes isocitrate dehydrogenase-dependent carboxylation of alpha-ketoglutarate to citrate to support cell growth and viability. Proc Natl Acad Sci U S A 2011; 108: 19611–19616. 22106302 10.1073/pnas.1117773108 PMC3241793

[40] Wise DR DeBerardinis RJ Mancuso A Myc regulates a transcriptional program that stimulates mitochondrial glutaminolysis and leads to glutamine addiction. Proc Natl Acad Sci U S A 2008; 105: 18782–18787. 19033189 10.1073/pnas.0810199105 PMC2596212

[41] Hosios AM Hecht VC Danai LV Amino acids rather than glucose account for the majority of cell mass in proliferating mammalian cells. Dev Cell 2016; 36: 540–549. 26954548 10.1016/j.devcel.2016.02.012 PMC4766004

[42] Haigis MC Deng CX Finley LW SIRT3 is a mitochondrial tumor suppressor: a scientific tale that connects aberrant cellular ROS, the Warburg effect, and carcinogenesis. Cancer Res 2012; 72: 2468–2472. 22589271 10.1158/0008-5472.CAN-11-3633 PMC3354726

[43] Finley LW Carracedo A Lee J SIRT3 opposes reprogramming of cancer cell metabolism through HIF1alpha destabilization. Cancer Cell 2011; 19: 416–428. 21397863 10.1016/j.ccr.2011.02.014 PMC3065720

[44] Paulin R Dromparis P Sutendra G Sirtuin 3 deficiency is associated with inhibited mitochondrial function and pulmonary arterial hypertension in rodents and humans. Cell Metab 2014; 20: 827–839. 25284742 10.1016/j.cmet.2014.08.011

[45] Lane KL Talati M Austin E Oxidative injury is a common consequence of BMPR2 mutations. Pulm Circ 2011; 1: 72–83. 21904662 10.4103/2045-8932.78107 PMC3167174

[46] Zhang R Sun ML Fan YF Plasma 15-F2t-isoprostane in idiopathic pulmonary arterial hypertension. Int J Cardiol 2014; 175: 268–273. 24877587 10.1016/j.ijcard.2014.05.014

[47] Monneret D Cracowski JL Bonnefont-Rousselot D Isoprostane as a promising prognostic biomarker in pulmonary arterial hypertension: preanalytical and analytical viewpoints. Int J Cardiol 2014; 177: 527–528. 25183532 10.1016/j.ijcard.2014.08.113

[48] Dromparis P Michelakis ED F2-isoprostanes: an emerging pulmonary arterial hypertension biomarker and potential link to the metabolic theory of pulmonary arterial hypertension? Chest 2012; 142: 816–820. 23032445 10.1378/chest.12-0848

[49] Cracowski JL Degano B Chabot F Independent association of urinary F2-isoprostanes with survival in pulmonary arterial hypertension. Chest 2012; 142: 869–876. 22406961 10.1378/chest.11-1267

[50] Diebold I Hennigs JK Miyagawa K BMPR2 preserves mitochondrial function and DNA during reoxygenation to promote endothelial cell survival and reverse pulmonary hypertension. Cell Metab 2015; 21: 596–608. 25863249 10.1016/j.cmet.2015.03.010 PMC4394191

[51] Fessel JP Hamid R Wittmann BM Metabolomic analysis of bone morphogenetic protein receptor type 2 mutations in human pulmonary endothelium reveals widespread metabolic reprogramming. Pulm Circ 2012; 2: 201–213. 22837861 10.4103/2045-8932.97606 PMC3401874

[52] Xu W Koeck T Lara AR Alterations of cellular bioenergetics in pulmonary artery endothelial cells. Proc Natl Acad Sci U S A 2007; 104: 1342–1347. 17227868 10.1073/pnas.0605080104 PMC1783136

[53] Lai YC Tabima DM Dube JJ SIRT3-AMP-activated protein kinase activation by nitrite and metformin improves hyperglycemia and normalizes pulmonary hypertension associated with heart failure with preserved ejection fraction. Circulation 2016; 133: 717–731. 26813102 10.1161/CIRCULATIONAHA.115.018935 PMC4766041

[54] Waypa GB Osborne SW Marks JD Sirtuin 3 deficiency does not augment hypoxia-induced pulmonary hypertension. Am J Respir Cell Mol Biol 2013; 49: 885–891. 24047466 10.1165/rcmb.2013-0191OC PMC3931121

[55] Nguyen TT Caito SW Zackert WE Scavengers of reactive gamma-ketoaldehydes extend Caenorhabditis elegans lifespan and healthspan through protein-level interactions with SIR-2.1 and ETS-7. Aging (Albany NY) 2016; 8: 1759–1780. 27514077 10.18632/aging.101011 PMC5032694

[56] Bertero T Oldham WM Cottrill KA Vascular stiffness mechanoactivates YAP/TAZ-dependent glutaminolysis to drive pulmonary hypertension. J Clin Invest 2016; 126: 3313–3335. 27548520 10.1172/JCI86387 PMC5004943

[57] Boulahbel H Duran RV Gottlieb E Prolyl hydroxylases as regulators of cell metabolism. Biochem Soc Trans 2009; 37: 291–294. 19143649 10.1042/BST0370291

[58] Selak MA Armour SM MacKenzie ED Succinate links TCA cycle dysfunction to oncogenesis by inhibiting HIF-alpha prolyl hydroxylase. Cancer Cell 2005; 7: 77–85. 15652751 10.1016/j.ccr.2004.11.022

[59] Schoors S Bruning U Missiaen R Fatty acid carbon is essential for dNTP synthesis in endothelial cells. Nature 2015; 520: 192–197. 25830893 10.1038/nature14362 PMC4413024

